# Quantitative metrics of bipolar electrograms predict ablation success in focal PVCs

**DOI:** 10.1002/joa3.70128

**Published:** 2025-06-30

**Authors:** Brijesh Sathian, Syed Muhammad Ali, Javed Iqbal, Ayesha Parvaiz Malik

**Affiliations:** ^1^ Hamad Medical Corporation Doha Qatar; ^2^ Jinnah Sindh Medical University Karachi Pakistan

Dear Editor,

We read with much interest the paper by Jeong et al. entitled “Quantitative analyses of the distal bipolar electrogram for focal premature ventricular contraction ablation”.^1^ The authors are to be welcomed for their effort to bring in objective electrogram parameters into a field long under the grip of empirical interpretation. Their application of t½, slope factor (S), and onset‐to‐surface ECG time (Ts) offers a robust statistical basis for the discrimination of near‐field vs. far‐field bipolar electrograms (bi‐EGMs) in catheter ablation of idiopathic PVCs. These parameters showed good discrimination using area under the curve (AUC) values greater than 0.85.

Yet, there are some points to note and areas of potential improvement. First, the retrospective single‐center study with just 41 cases could be a drawback regarding external generalizability. Prospective multicenter validation with different operators and variable anatomical complexity is called for. Second, though the authors excluded unipolar EGM analysis owing to previously reported limitations,^2^ comparative analysis would have given interesting insight into the incremental value of bipolar quantification.

The incorporation of these parameters into electroanatomic mapping platforms also poses questions regarding feasibility. For instance, whereas t½ and Ts are easy to compute, the calculation of the slope factor S through nonlinear regression might not be practical in real‐time without software development or adaptation.^3^ Further, while the authors propose Ts values can be used to predict lesion depth (~4 mm), this presumption must be histologically validated since lesion depth is also determined by catheter force, duration, and tissue thickness.^4^

Another noteworthy point is that the count of deflections (De#), a venerable target in scar‐based VT ablation, lacked predictive ability regarding ablation success in this series. This contradicts common practices and favors a move toward sharper and earlier bi‐EGM features as better indicators of effective lesion targeting. But fractionation morphology analysis instead of mere deflection count may provide greater insight into future work.^5^

Despite such restraints, the study is a valuable step toward standardization of EGM interpretation and could potentially have important clinical applications if used in conjunction with real‐time mapping systems. We would support further multicenter prospective investigations and further development of automated tools to compute these parameters during ablation (Table 1).

**TABLE 1:** Summary of Electrogram Parameters and Their Predictive Utility for Successful Ablation (Adapted From Jeong et al.^1^).

Parameter	Successful ablation (mean ± SD)	Unsuccessful ablation (mean ± SD)	*p*‐value	AUC (ROC)	Optimal cutoff	Sensitivity (%)	Specificity (%)
Half time of activation (t½, ms)	3.18 ± 0.25	5.85 ± 0.64	<0.001	0.85	5.0 ms	92.3	73.3
Slope factor (S, ms)	0.82 ± 0.09	4.83 ± 2.01	0.01	0.88	1.8 ms	92.3	66.7
Time to surface ECG (Ts, ms)	35.62 ± 1.25	25.80 ± 1.64	<0.001	0.87	33 ms	92.3	66.7
Linear slope (dV/dt, mV/ms)	0.16 ± 0.04	0.05 ± 0.02	0.06	0.76	—	—	—
Number of deflections (De#)	2.65 ± 0.22	2.27 ± 0.28	0.29	0.60	—	—	—

## CONFLICT OF INTEREST STATEMENT

The authors declare no conflicts of interest.
